# 3-(3-Chloro­phenyl­sulfin­yl)-2,4,6,7-tetra­methyl-1-benzofuran

**DOI:** 10.1107/S1600536812002887

**Published:** 2012-01-31

**Authors:** Hong Dae Choi, Pil Ja Seo, Uk Lee

**Affiliations:** a Department of Chemistry, Dongeui University, San 24 Kaya-dong Busanjin-gu, Busan 614-714, Republic of Korea; b Department of Chemistry, Pukyong National University, 599-1 Daeyeon 3-dong, Nam-gu, Busan 608-737, Republic of Korea

## Abstract

In the title compound, C_18_H_17_ClO_2_S, the 3-chloro­phenyl ring makes a dihedral angle of 72.62 (4)° with the mean plane of the benzofuran fragment. In the crystal, mol­ecules are linked by weak inter­molecular C—H⋯O and C—H⋯π inter­actions. The crystal structure also exhibits a slipped π–π inter­action between the 3-chloro­phenyl rings of adjacent mol­ecules [centroid–centroid distance = 3.751 (2) Å, inter­planar distance = 3.450 (2) Å and slippage = 1.472 (2) Å].

## Related literature

For the pharmacological activity of benzofuran compounds, see: Aslam *et al.* (2009 ▶); Galal *et al.* (2009 ▶); Khan *et al.* (2005 ▶). For natural products with benzofuran rings, see: Akgul & Anil (2003 ▶); Soekamto *et al.* (2003 ▶). For the crystal structures of related compounds, see: Choi *et al.* (2010 ▶, 2011 ▶).
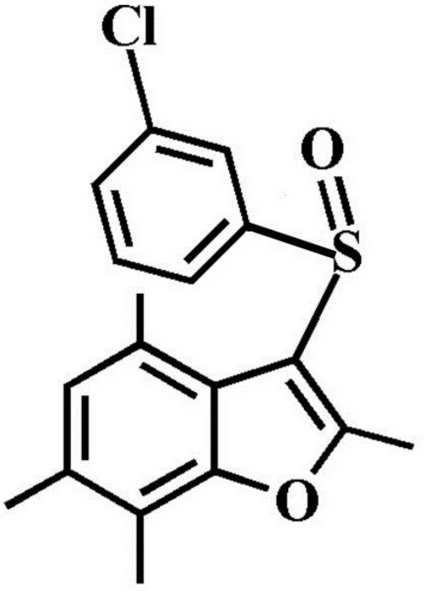


## Experimental

### 

#### Crystal data

C_18_H_17_ClO_2_S
*M* *_r_* = 332.83
Triclinic, 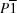

*a* = 7.4198 (1) Å
*b* = 7.9792 (1) Å
*c* = 14.5014 (2) Å
α = 105.965 (1)°
β = 95.732 (1)°
γ = 103.965 (1)°
*V* = 788.21 (2) Å^3^
*Z* = 2
Mo *K*α radiation
μ = 0.38 mm^−1^
*T* = 173 K
0.26 × 0.25 × 0.25 mm

#### Data collection

Bruker SMART APEXII CCD diffractometer
Absorption correction: multi-scan (*SADABS*; Bruker, 2009 ▶) *T*_min_ = 0.908, *T*_max_ = 0.911
14786 measured reflections
3897 independent reflections
3622 reflections with *I* > 2σ(*I*)
*R*_int_ = 0.025

#### Refinement

*R*[*F* ^2^ > 2σ(*F* ^2^)] = 0.035
*wR*(*F* ^2^) = 0.099
*S* = 0.95
3897 reflections
203 parameters
H-atom parameters constrained
Δρ_max_ = 0.36 e Å^−3^
Δρ_min_ = −0.39 e Å^−3^

### 

Data collection: *APEX2* (Bruker, 2009 ▶); cell refinement: *SAINT* (Bruker, 2009 ▶); data reduction: *SAINT*; program(s) used to solve structure: *SHELXS97* (Sheldrick, 2008 ▶); program(s) used to refine structure: *SHELXL97* (Sheldrick, 2008 ▶); molecular graphics: *ORTEP-3* (Farrugia, 1997 ▶) and *DIAMOND* (Brandenburg, 1998 ▶); software used to prepare material for publication: *SHELXL97*.

## Supplementary Material

Crystal structure: contains datablock(s) global, I. DOI: 10.1107/S1600536812002887/ds2170sup1.cif

Structure factors: contains datablock(s) I. DOI: 10.1107/S1600536812002887/ds2170Isup2.hkl

Supplementary material file. DOI: 10.1107/S1600536812002887/ds2170Isup3.cml

Additional supplementary materials:  crystallographic information; 3D view; checkCIF report

## Figures and Tables

**Table 1:** Hydrogen-bond geometry (Å, °) *Cg*1 is the centroid of the C2–C7 benzene ring.

*D*—H⋯*A*	*D*—H	H⋯*A*	*D*⋯*A*	*D*—H⋯*A*
C11—H11*B*⋯O2^i^	0.98	2.60	3.5786 (16)	175
C12—H12*A*⋯O2^ii^	0.98	2.40	3.3159 (17)	155
C10—H10*C*⋯*Cg*1^iii^	0.98	2.79	3.615 (16)	142

Symmetry codes: (i) 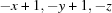; (ii) 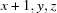; (iii) 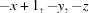.

**Fig. 1.**
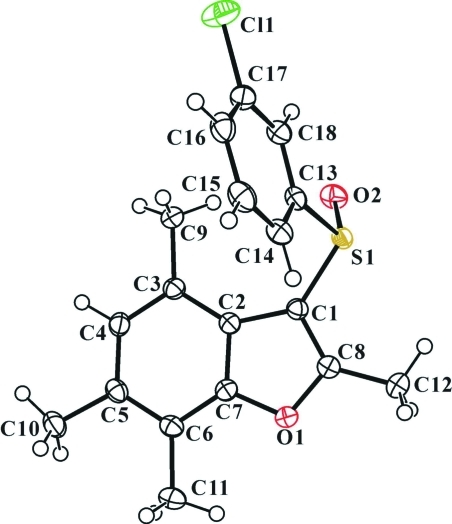
The molecular structure of the title compound with the atom numbering scheme. Displacement ellipsoids are drawn at the 50% probability level. H atoms are presented as small spheres of arbitrary radius.

**Fig. 2.**
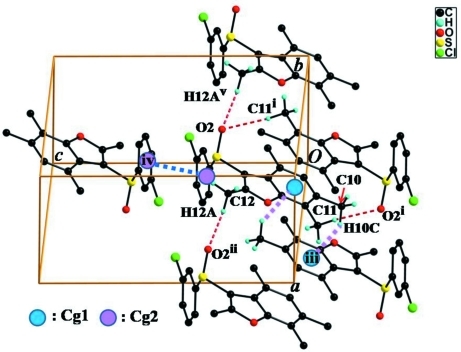
A view of the C–H···O, C–H···π, and π–π interactions (dottedlines) in the crystal structure of the title compound. H atoms non-participating in hydrogen-bonding were omitted for clarity. [Symmetry codes: (i) - x + 1, - y + 1, - z; (ii) x + 1, y, z; (iii) -x + 1, -y, -z (iv) - -x + 1, - -y + 1, - z + 1; (v) -x - 1, -y, z.]

## supplementary crystallographic information

### Comment

Substituted benzofuran analogues have drawn much attention owing to their valuable biological properties such as antibacterial and antifungal, antitumor and antiviral, and antimicrobial activities (Aslam *et al.*, 2009, Galal *et al.*, 2009, Khan *et al.*, 2005). These benzofuran derivatives occur in a wide range of natural products (Akgul & Anil, 2003; Soekamto *et al.*, 2003). As a part of our continuing study of 2,4,6,7-tetramethyl-1-benzofuran derivatives containing either 3-(4-chlorophenylsulfinyl) (Choi *et al.*, 2010) or 3-(3-fluorophenylsulfinyl) (Choi *et al.*, 2011) substituents, we report herein the crystal structure of the title compound.

In the title molecule (Fig. 1), the benzofuran unit is essentially planar, with a mean deviation of 0.018 (1) Å from the least-squares plane defined by the nine constituent atoms. The dihedral angle between the 3-chlorophenyl ring and the mean plane of the benzofurn fragment is 72.62 (4)°. The crystal packing (Fig. 2) is stabilized by weak intermolecular C– H···O and C–H···π interactions (Table 1, Cg1 is the centroid of the C2-C7 benzene ring). The crystal packing (Fig. 2) is further stabilized by a weak slipped π–π interaction between the 3-chlorophenyl rings of adjacent molecules, with a Cg2···Cg2^iv^ distance of 3.751 (2) Å and an interplanar distance of 3.450 (2) Å resulting in a slippage of 1.472 (2) Å (Cg2 is the centroid of the C13-C18 3-chlorophenyl ring).

### Experimental

77% 3-chloroperoxybenzoic acid (224 mg, 1.0 mmol) was added in small portions to a stirred solution of 3-(3-chlorophenylsulfanyl)-2,4,6,7-tetramethyl-1-benzofuran (285 mg, 0.9 mmol) in dichloromethane (30 mL) at 273 K. After being stirred at room temperature for 10h, the mixture was washed with saturated sodium bicarbonate solution and the organic layer was separated, dried over magnesium sulfate, filtered and concentrated at reduced pressure. The residue was purified by column chromatography (hexane-ethyl acetate, 4:1 v/v) to afford the title compound as a colorless solid [yield 76%, m.p. 447-448 K; Rf = 0.52 (hexane-ethyl acetate, 4:1 v/v)]. Single crystals suitable for X-ray diffraction were prepared by slow evaporation of a solution of the title compound in ethyl acetate at room temperature

### Refinement

All H atoms were positioned geometrically and refined using a riding model, with C–H = 0.95 Å for aryl and 0.98 Å for methyl H atoms. *U*_iso_(H) =1.2*U*_eq_(C) for aryl and 1.5*U*_eq_(C)for methyl H atoms.

### Crystal data

**Table:**

C_18_H_17_ClO_2_S	*Z* = 2
*M**_r_* = 332.83	*F*(000) = 348
Triclinic, *P*1	*D*_x_ = 1.402 Mg m^−^^3^
Hall symbol: -P 1	Mo *K*α radiation, λ = 0.71073 Å
*a* = 7.4198 (1) Å	Cell parameters from 9057 reflections
*b* = 7.9792 (1) Å	θ = 2.7–28.3°
*c* = 14.5014 (2) Å	µ = 0.38 mm^−^^1^
α = 105.965 (1)°	*T* = 173 K
β = 95.732 (1)°	Block, colourless
γ = 103.965 (1)°	0.26 × 0.25 × 0.25 mm
*V* = 788.21 (2) Å^3^	

### Data collection

**Table:**

Bruker SMART APEXII CCD diffractometer	3897 independent reflections
Radiation source: rotating anode	3622 reflections with *I* > 2σ(*I*)
graphite multilayer	*R*_int_ = 0.025
Detector resolution: 10.0 pixels mm^-1^	θ_max_ = 28.3°, θ_min_ = 1.5°
φ and ω scans	*h* = −9→9
Absorption correction: multi-scan (*SADABS*; Bruker, 2009)	*k* = −10→10
*T*_min_ = 0.908, *T*_max_ = 0.911	*l* = −19→19
14786 measured reflections	

### Refinement

**Table:**

Refinement on *F*^2^	Primary atom site location: structure-invariant direct methods
Least-squares matrix: full	Secondary atom site location: difference Fourier map
*R*[*F*^2^ > 2σ(*F*^2^)] = 0.035	Hydrogen site location: difference Fourier map
*wR*(*F*^2^) = 0.099	H-atom parameters constrained
*S* = 0.95	*w* = 1/[σ^2^(*F*_o_^2^) + (0.0638*P*)^2^ + 0.3249*P*] where *P* = (*F*_o_^2^ + 2*F*_c_^2^)/3
3897 reflections	(Δ/σ)_max_ = 0.001
203 parameters	Δρ_max_ = 0.36 e Å^−^^3^
0 restraints	Δρ_min_ = −0.39 e Å^−^^3^

### Special details

**Table:**

Geometry. All esds (except the esd in the dihedral angle between two l.s. planes) are estimated using the full covariance matrix. The cell esds are taken into account individually in the estimation of esds in distances, angles and torsion angles; correlations between esds in cell parameters are only used when they are defined by crystal symmetry. An approximate (isotropic) treatment of cell esds is used for estimating esds involving l.s. planes.
Refinement. Refinement of F^2^ against ALL reflections. The weighted R-factor wR and goodness of fit S are based on F^2^, conventional R-factors R are based on F, with F set to zero for negative F^2^. The threshold expression of F^2^ > 2sigma(F^2^) is used only for calculating R-factors(gt) etc. and is not relevant to the choice of reflections for refinement. R-factors based on F^2^ are statistically about twice as large as those based on F, and R- factors based on ALL data will be even larger.

### Fractional atomic coordinates and isotropic or equivalent isotropic displacement parameters (Å^2^)

**Table:**

	*x*	*y*	*z*	*U*_iso_*/*U*_eq_	
S1	0.53130 (4)	0.65049 (4)	0.33172 (2)	0.02082 (9)	
Cl1	−0.00317 (6)	0.23558 (6)	0.46616 (3)	0.04572 (13)	
O1	0.79660 (12)	0.52179 (12)	0.11618 (6)	0.02335 (19)	
O2	0.36498 (14)	0.72242 (13)	0.32004 (7)	0.0282 (2)	
C1	0.59030 (17)	0.55512 (16)	0.21874 (8)	0.0202 (2)	
C2	0.48969 (16)	0.40937 (15)	0.13006 (8)	0.0191 (2)	
C3	0.30551 (17)	0.29506 (16)	0.09430 (9)	0.0212 (2)	
C4	0.27547 (18)	0.17027 (17)	0.00165 (9)	0.0236 (2)	
H4	0.1518	0.0915	−0.0247	0.028*	
C5	0.41627 (19)	0.15371 (17)	−0.05513 (9)	0.0232 (2)	
C6	0.59915 (18)	0.26984 (17)	−0.02096 (9)	0.0220 (2)	
C7	0.62560 (17)	0.39462 (16)	0.07062 (8)	0.0204 (2)	
C8	0.77070 (17)	0.61729 (17)	0.20508 (9)	0.0227 (2)	
C9	0.14666 (18)	0.30115 (19)	0.15074 (9)	0.0270 (3)	
H9A	0.0265	0.2612	0.1053	0.040*	
H9B	0.1654	0.4257	0.1926	0.040*	
H9C	0.1445	0.2205	0.1911	0.040*	
C10	0.3716 (2)	0.00812 (19)	−0.15260 (9)	0.0297 (3)	
H10A	0.4041	0.0640	−0.2033	0.045*	
H10B	0.2366	−0.0561	−0.1674	0.045*	
H10C	0.4450	−0.0781	−0.1504	0.045*	
C11	0.7563 (2)	0.26059 (19)	−0.07831 (10)	0.0285 (3)	
H11A	0.8681	0.3618	−0.0442	0.043*	
H11B	0.7166	0.2685	−0.1431	0.043*	
H11C	0.7866	0.1454	−0.0850	0.043*	
C12	0.93714 (19)	0.7651 (2)	0.26499 (10)	0.0317 (3)	
H12A	1.0409	0.7140	0.2781	0.048*	
H12B	0.9046	0.8265	0.3268	0.048*	
H12C	0.9761	0.8526	0.2298	0.048*	
C13	0.44562 (18)	0.44638 (16)	0.36349 (8)	0.0211 (2)	
C14	0.55149 (19)	0.32357 (18)	0.35979 (9)	0.0264 (3)	
H14	0.6695	0.3438	0.3384	0.032*	
C15	0.4822 (2)	0.17083 (18)	0.38788 (10)	0.0305 (3)	
H15	0.5528	0.0854	0.3851	0.037*	
C16	0.3106 (2)	0.14177 (18)	0.42004 (10)	0.0298 (3)	
H16	0.2625	0.0365	0.4385	0.036*	
C17	0.21090 (19)	0.26868 (19)	0.42478 (9)	0.0276 (3)	
C18	0.27641 (18)	0.42257 (18)	0.39725 (9)	0.0246 (3)	
H18	0.2069	0.5092	0.4015	0.030*	

### Atomic displacement parameters (Å^2^)

**Table:**

	*U*^11^	*U*^22^	*U*^33^	*U*^12^	*U*^13^	*U*^23^
S1	0.02414 (16)	0.01806 (15)	0.01927 (15)	0.00564 (11)	0.00480 (11)	0.00411 (11)
Cl1	0.0391 (2)	0.0496 (2)	0.0587 (3)	0.01101 (18)	0.02486 (18)	0.0286 (2)
O1	0.0202 (4)	0.0255 (4)	0.0230 (4)	0.0046 (3)	0.0058 (3)	0.0062 (3)
O2	0.0326 (5)	0.0247 (5)	0.0331 (5)	0.0151 (4)	0.0106 (4)	0.0104 (4)
C1	0.0211 (5)	0.0205 (5)	0.0193 (5)	0.0056 (4)	0.0043 (4)	0.0063 (4)
C2	0.0224 (6)	0.0177 (5)	0.0187 (5)	0.0062 (4)	0.0044 (4)	0.0074 (4)
C3	0.0224 (6)	0.0204 (5)	0.0217 (5)	0.0056 (4)	0.0054 (4)	0.0080 (4)
C4	0.0243 (6)	0.0204 (6)	0.0228 (6)	0.0022 (5)	0.0025 (4)	0.0055 (5)
C5	0.0306 (6)	0.0207 (6)	0.0190 (5)	0.0080 (5)	0.0049 (4)	0.0064 (4)
C6	0.0274 (6)	0.0222 (6)	0.0205 (5)	0.0093 (5)	0.0077 (4)	0.0096 (4)
C7	0.0207 (5)	0.0205 (5)	0.0214 (5)	0.0057 (4)	0.0040 (4)	0.0087 (4)
C8	0.0232 (6)	0.0231 (6)	0.0220 (5)	0.0069 (5)	0.0046 (4)	0.0068 (5)
C9	0.0215 (6)	0.0293 (6)	0.0255 (6)	0.0021 (5)	0.0064 (5)	0.0046 (5)
C10	0.0387 (7)	0.0249 (6)	0.0219 (6)	0.0073 (5)	0.0064 (5)	0.0027 (5)
C11	0.0311 (7)	0.0330 (7)	0.0247 (6)	0.0115 (5)	0.0118 (5)	0.0097 (5)
C12	0.0217 (6)	0.0333 (7)	0.0315 (7)	0.0010 (5)	0.0034 (5)	0.0027 (6)
C13	0.0265 (6)	0.0195 (5)	0.0163 (5)	0.0066 (4)	0.0034 (4)	0.0039 (4)
C14	0.0307 (6)	0.0254 (6)	0.0246 (6)	0.0113 (5)	0.0069 (5)	0.0066 (5)
C15	0.0421 (8)	0.0235 (6)	0.0288 (6)	0.0147 (6)	0.0068 (6)	0.0075 (5)
C16	0.0403 (7)	0.0216 (6)	0.0247 (6)	0.0040 (5)	0.0042 (5)	0.0075 (5)
C17	0.0294 (6)	0.0285 (6)	0.0228 (6)	0.0036 (5)	0.0064 (5)	0.0078 (5)
C18	0.0282 (6)	0.0258 (6)	0.0207 (6)	0.0088 (5)	0.0057 (5)	0.0070 (5)

### Geometric parameters (Å, °)

**Table:**

S1—O2	1.4949 (9)	C9—H9C	0.9800
S1—C1	1.7531 (12)	C10—H10A	0.9800
S1—C13	1.8001 (13)	C10—H10B	0.9800
Cl1—C17	1.7401 (14)	C10—H10C	0.9800
O1—C8	1.3635 (14)	C11—H11A	0.9800
O1—C7	1.3851 (14)	C11—H11B	0.9800
C1—C8	1.3651 (17)	C11—H11C	0.9800
C1—C2	1.4594 (16)	C12—H12A	0.9800
C2—C7	1.3960 (16)	C12—H12B	0.9800
C2—C3	1.4018 (17)	C12—H12C	0.9800
C3—C4	1.3941 (17)	C13—C18	1.3823 (17)
C3—C9	1.5032 (16)	C13—C14	1.3897 (17)
C4—C5	1.4020 (17)	C14—C15	1.3886 (19)
C4—H4	0.9500	C14—H14	0.9500
C5—C6	1.3970 (18)	C15—C16	1.388 (2)
C5—C10	1.5081 (17)	C15—H15	0.9500
C6—C7	1.3856 (17)	C16—C17	1.382 (2)
C6—C11	1.5025 (17)	C16—H16	0.9500
C8—C12	1.4797 (18)	C17—C18	1.3870 (19)
C9—H9A	0.9800	C18—H18	0.9500
C9—H9B	0.9800		
			
O2—S1—C1	111.44 (6)	C5—C10—H10B	109.5
O2—S1—C13	105.69 (6)	H10A—C10—H10B	109.5
C1—S1—C13	99.12 (5)	C5—C10—H10C	109.5
C8—O1—C7	106.80 (9)	H10A—C10—H10C	109.5
C8—C1—C2	107.14 (10)	H10B—C10—H10C	109.5
C8—C1—S1	118.34 (9)	C6—C11—H11A	109.5
C2—C1—S1	134.45 (9)	C6—C11—H11B	109.5
C7—C2—C3	118.68 (11)	H11A—C11—H11B	109.5
C7—C2—C1	104.27 (10)	C6—C11—H11C	109.5
C3—C2—C1	137.04 (11)	H11A—C11—H11C	109.5
C4—C3—C2	116.04 (11)	H11B—C11—H11C	109.5
C4—C3—C9	120.50 (11)	C8—C12—H12A	109.5
C2—C3—C9	123.45 (11)	C8—C12—H12B	109.5
C3—C4—C5	124.13 (12)	H12A—C12—H12B	109.5
C3—C4—H4	117.9	C8—C12—H12C	109.5
C5—C4—H4	117.9	H12A—C12—H12C	109.5
C6—C5—C4	120.18 (11)	H12B—C12—H12C	109.5
C6—C5—C10	119.76 (11)	C18—C13—C14	121.49 (12)
C4—C5—C10	120.05 (12)	C18—C13—S1	117.34 (9)
C7—C6—C5	114.84 (11)	C14—C13—S1	121.06 (10)
C7—C6—C11	122.20 (12)	C15—C14—C13	118.97 (12)
C5—C6—C11	122.96 (11)	C15—C14—H14	120.5
O1—C7—C6	123.21 (11)	C13—C14—H14	120.5
O1—C7—C2	110.72 (10)	C16—C15—C14	120.63 (12)
C6—C7—C2	126.06 (11)	C16—C15—H15	119.7
O1—C8—C1	111.04 (11)	C14—C15—H15	119.7
O1—C8—C12	115.43 (11)	C17—C16—C15	118.86 (13)
C1—C8—C12	133.53 (12)	C17—C16—H16	120.6
C3—C9—H9A	109.5	C15—C16—H16	120.6
C3—C9—H9B	109.5	C16—C17—C18	121.86 (13)
H9A—C9—H9B	109.5	C16—C17—Cl1	119.46 (11)
C3—C9—H9C	109.5	C18—C17—Cl1	118.68 (11)
H9A—C9—H9C	109.5	C13—C18—C17	118.15 (12)
H9B—C9—H9C	109.5	C13—C18—H18	120.9
C5—C10—H10A	109.5	C17—C18—H18	120.9
			
O2—S1—C1—C8	125.23 (10)	C11—C6—C7—C2	−178.00 (11)
C13—S1—C1—C8	−123.83 (10)	C3—C2—C7—O1	177.83 (10)
O2—S1—C1—C2	−58.15 (13)	C1—C2—C7—O1	−1.15 (12)
C13—S1—C1—C2	52.78 (13)	C3—C2—C7—C6	−3.17 (18)
C8—C1—C2—C7	1.49 (13)	C1—C2—C7—C6	177.85 (11)
S1—C1—C2—C7	−175.39 (10)	C7—O1—C8—C1	0.63 (13)
C8—C1—C2—C3	−177.20 (13)	C7—O1—C8—C12	−178.36 (11)
S1—C1—C2—C3	5.9 (2)	C2—C1—C8—O1	−1.34 (14)
C7—C2—C3—C4	1.96 (16)	S1—C1—C8—O1	176.13 (8)
C1—C2—C3—C4	−179.49 (13)	C2—C1—C8—C12	177.39 (14)
C7—C2—C3—C9	−178.71 (11)	S1—C1—C8—C12	−5.1 (2)
C1—C2—C3—C9	−0.2 (2)	O2—S1—C13—C18	−17.61 (11)
C2—C3—C4—C5	0.39 (18)	C1—S1—C13—C18	−133.06 (10)
C9—C3—C4—C5	−178.96 (12)	O2—S1—C13—C14	166.23 (10)
C3—C4—C5—C6	−1.86 (19)	C1—S1—C13—C14	50.79 (11)
C3—C4—C5—C10	177.04 (12)	C18—C13—C14—C15	2.02 (19)
C4—C5—C6—C7	0.82 (17)	S1—C13—C14—C15	178.01 (10)
C10—C5—C6—C7	−178.09 (11)	C13—C14—C15—C16	−0.6 (2)
C4—C5—C6—C11	−179.50 (11)	C14—C15—C16—C17	−0.8 (2)
C10—C5—C6—C11	1.59 (18)	C15—C16—C17—C18	0.8 (2)
C8—O1—C7—C6	−178.65 (11)	C15—C16—C17—Cl1	−179.31 (10)
C8—O1—C7—C2	0.39 (13)	C14—C13—C18—C17	−2.01 (18)
C5—C6—C7—O1	−179.43 (10)	S1—C13—C18—C17	−178.15 (9)
C11—C6—C7—O1	0.88 (18)	C16—C17—C18—C13	0.59 (19)
C5—C6—C7—C2	1.68 (18)	Cl1—C17—C18—C13	−179.31 (9)

### Hydrogen-bond geometry (Å, °)

**Table:**

Cg1 is the centroid of the C2–C7 benzene ring.

**Table:**

*D*—H···*A*	*D*—H	H···*A*	*D*···*A*	*D*—H···*A*
C11—H11B···O2^i^	0.98	2.60	3.5786 (16)	175
C12—H12A···O2^ii^	0.98	2.40	3.3159 (17)	155
C10—H10C···Cg1^iii^	0.98	2.79	3.615 (16)	142

Symmetry codes: (i) −*x*+1, −*y*+1, −*z*; (ii) *x*+1, *y*, *z*; (iii) −*x*+1, −*y*, −*z*.
